# Molecular Approaches for Detection of *Trichoderma* Green Mold Disease in Edible Mushroom Production

**DOI:** 10.3390/biology12020299

**Published:** 2023-02-14

**Authors:** Ljiljana Šašić Zorić, Ljiljana Janjušević, Mila Djisalov, Teodora Knežić, Jovana Vunduk, Ivanka Milenković, Ivana Gadjanski

**Affiliations:** 1 BioSense Institute, University of Novi Sad, Dr Zorana Đinđića 1, 21000 Novi Sad, Serbia; 2 Institute of General and Physical Chemistry, University of Belgrade, Studentski trg 12/V, 11080 Belgrade, Serbia; 3 EKOFUNGI DOO, Zrenjaninski put bb, Industrijska zona, Padinska Skela, 11213 Belgrade, Serbia

**Keywords:** edible mushrooms, green mold, in-field detection, molecular diagnostics, point-of-need devices, *Trichoderma*

## Abstract

**Simple Summary:**

The green mold disease caused by the pathogenic fungi *Trichoderma* spp. is the most harmful disease for edible mushroom production. This disease’s harmful effect is due to the belated detection of the green mold disease, which occurs when the damage to the yield is irreversible. Severe epidemics of green mold started during the 1980s and 1990s in Europe and America and triggered the development of molecular approaches for the monitoring and detection of *Trichoderma*. The most promising molecular tools are nucleic acid (NA)-based methods. In this review, we discuss the currently most-used molecular methods for green mold detection and introduce an NA-based isothermal amplification methodology suitable for the development of point-of-need (PON) devices for field applications in detecting this disease.

**Abstract:**

Due to the evident aggressive nature of green mold and the consequently huge economic damage it causes for producers of edible mushrooms, there is an urgent need for prevention and infection control measures, which should be based on the early detection of various *Trichoderma* spp. as green mold causative agents. The most promising current diagnostic tools are based on molecular methods, although additional optimization for real-time, in-field detection is still required. In the first part of this review, we briefly discuss cultivation-based methods and continue with the secondary metabolite-based methods. Furthermore, we present an overview of the commonly used molecular methods for *Trichoderma* species/strain detection. Additionally, we also comment on the potential of genomic approaches for green mold detection. In the last part, we discuss fast screening molecular methods for the early detection of *Trichoderma* infestation with the potential for in-field, point-of-need (PON) application, focusing on isothermal amplification methods. Finally, current challenges and future perspectives in *Trichoderma* diagnostics are summarized in the conclusions.

## 1. Introduction

There are numerous mushroom species that can be used as a food source. Edible mushrooms can be considered healthy food with low calorific value and low content of fat but rich in proteins, dietary fibers and minerals. Their nutritional value, antioxidant activity, and therapeutic properties, as well as their flavor and unique texture, make them attractive for use as a food ingredient or additive replacer in foods [1,2,3]. The most widely cultivated edible mushrooms are *Lentinula edodes* (shiitake), *Auricularia auricula* (wood ear), *Pleurotus ostreatus* (oyster mushroom) and *Agaricus bisporus* (champignon) [4,5,6].

Edible mushroom production is strongly affected by the presence of fungal pathogens *Trichoderma* spp. that are causative agents of green mold disease, although it can be caused by *Penicillium* and *Aspergillus*, other green molds with weak pathogenicity. *Trichoderma* initially produces white and dense mycelia that change color during sporulation and finally become dark green and visible on mushroom-growing substrate. It also causes the appearance of brown spots of necrotic tissue and lesions on mushroom fruiting bodies [7]. More than 30 *Trichoderma* species are pathogens of mushrooms and have been found in the substrate and fruiting bodies of *A. bisporus*, *L. edodes*, *P. ostreatus*, *Ganoderma lingzhi*, etc. [8,9].

Button mushroom or champignon, *Agaricus bisporus,* is the fourth most-produced mushroom species in the world and accounts for 15% of world total mushroom production, with China, the USA, Poland, the Netherlands and India as its biggest producers [4,5,6]. Since green mold disease in *A. bisporus* production can cause significant yield losses, ranging from 60% to 100%, disease outbreaks may lead to serious economic costs for the producers. The disease is characterized by a rapid infestation of the compost by *Trichoderma* spp. and the subsequent inhibition of *A. bisporus* fructification. On the one hand, infection with *Trichoderma* causes lower yield or no fructification, and on the other hand, it also causes malformation of the fruit bodies [10,11,12]. In the case of the latter, all parameters of quality are affected. Infected white button mushrooms are small and abnormally shaped with lesions and dark-brown discoloration. The hat weight and diameter, as well as stipe weight, diameter and length, are also influenced [13].

*Agaricus bisporus* was first cultivated over 350 years ago and, at the time, was more resilient to the green mold; hence, the disease caused only minor crop losses for growers until the 1980s [14,15,16]. In the 1980s, a severe epidemic of green mold caused crop losses in the British Isles [17,18,19,20,21,22,23,24], while in the early 1990s, a similar epidemic happened in Ontario [25], British Columbia [25] and Pennsylvania [26]. The estimated economic damage exceeded USD 30 million in North America alone [16].

Severe epidemics of green mold disease on *A. bisporus* mushroom farms in Europe and USA during the 1980s and 1990s of the 20th century were related to aggressive *T. harzianum* strains or biotypes Th2 and Th4 [27,28], and some authors even suspected it to be due to the release of some *T. harzianum* biocontrol strains [16]. Moreover, researchers suggested that aggressive biotypes could, in fact, represent separate species based on DNA analyses [27], while Samuels et al. [29] finally described Th4 and Th2 biotypes as two forms of the new *Trichoderma* species *T. aggressivum* f. *aggressivum* and *T. aggressivum* f. *europaeum*. The description followed the findings of several molecular studies [16,27,28,29,30,31].

In addition to *A. bisporus*, green mold disease was reported on other commercially cultivated mushroom species such as *L. edodes*, *Pleurotus* spp., and recently on the medicinal mushroom *G. lingzhi* (lingzhi mushroom), making the economic impact of the disease even greater [32,33,34]. Park et al. [35] described two new *Trichoderma* species associated with the green mold epidemics of commercially grown oyster mushrooms in Korea, *T. pleuroti* (synonym: *T. pleurotum*) and *T. pleuroticola*, while Jayalal and Adikaram [36] reported that *Trichoderma harzianum* accounts for the loss of 20% of all oyster mushrooms produced in Sri Lanka. Additionally, *T. hengshanicum* and *T. atroviride* developing on *G. lingzhi* covered fruit bodies with green mycelium, causing lesions, rotting and moldering, which made the final product unacceptable for the market [32,37]. Chen et al. [38] reported *T. koningiopsis* green mold in *Dictyophora rubrovolvata*, while Cao et al. [8] reported three new *Trichoderma* species (*T. auriculariae*, *T. miyunense* and *T. pholiotae*) in the contaminated substrate of edible fungi.

Green mold can be easily spread by contaminated tools, substrate and even clothing of mushroom growers. It can also be spread by contaminated air and insect vectors (sciarid mushroom flies). Thus, strict hygiene, treatments with disinfectants and applications of fungicides are necessary to minimize contamination in mushroom farms [7,39,40]. Among fungicides, prochloraz and metrafenone proved to be effective against strains of various taxa of the genus *Trichoderma* (*T. harzianum* species complex, *T. aggressivum* f. *europaeum*, *T. pleuroti*, and *T. pleuroticola*) isolated from cultivations of edible mushrooms [7]. Additionally, with the rise of organic farming, environmentally friendly alternative methods such as the application of plant essential oils (e.g., basil and mint oil) and biocontrol based on microorganisms (mostly *Bacillus* species) have become more popular [39,40].

Given the aggressive nature of green mold and the huge economic damage as a consequence, there is an urgent need for prevention and infection control measures, which should be based on the early detection of *Trichoderma* spp. that cause green mold disease. In addition to traditional methods based on detection in culture, great importance has recently been given to molecular methods. The popularity of molecular over cultivation-based methods is connected to the ability of molecular tools to unambiguously detect *Trichoderma* species or even a particular strain causing green mold in the mushroom-growing substrate. Some of these methods can be adapted for fast turn-around time in-field application at point-of-need (PON). In light of these perspectives, the aim of this review is to give an overview of previously established and new molecular methods that can be used to detect *Trichoderma* infestation during mushroom production. More specifically, it includes (1) a historical overview of traditional detection methods, (2) a review of molecular diagnostic methods previously applied to *Trichoderma* spp. causing green mold, and (3) an exploration of new methods applied to other fungi with promising potential for *Trichoderma* green mold diagnosis.

## 2. Culture-Based Methods to Detect *Trichoderma* spp.

The conventional approach for the isolation and identification of *Trichoderma* spp. involves culturing on general or selective media, followed by observation of the macro- and micro-morphological characteristics. Although cheap and easy to perform, this approach has several disadvantages. The most important is that this culturing method is time-consuming, often taking several days to weeks due to the long generation time of fungi. In genera such as *Trichoderma*, visual characteristics of the reproductive structures, important for identification, are the last to be formed in culture. *Trichoderma*’s vegetative part, mycelium, is indistinguishable from the mycelium of *A. bisporus* [41]. The same applies to *Pleurotus* species [42]. Being almost the same in many species, the mycelium gives a restricted amount of diagnostic information. The aggressor is only recognizable when it is too late; sporulation is abundant, and small, colored and light-weighted spores are easily dispersed in the surrounding. Under production circumstances, such a time frame leads to yield and economic losses or even an epidemic.

As observed in medical practice, sample collecting and transport are of high importance when invasive species are being examined [43]. In mushroom cultivation, this step means that pieces of compost/substrate are transferred to a laboratory to be further cultivated. These samples are complex and naturally contain different microorganisms, especially bacteria, as well as other fungi, which might aggravate the isolation of pure culture. In practical terms, this means that suspected fungi need to be retransferred on new culture media and cultivated again. Occasionally, this step needs to be repeated several times until a pure culture of the suspected fungus is obtained. Concerning the growth media, *Trichoderma* spp. growth is supported by standard media, such as potato dextrose agar or malt agar, as well as specific media, such as *Trichoderma* selective medium, which was developed for the isolation of *Trichoderma* originating from soil [44]. Similarly, Williams et al. [45] evaluated the suitability of different growing media for the isolation of *Trichoderma* spp. from soil and found modified potato dextrose agar with rose bengal, chloramphenicol and streptomycin to be the most suitable. However, media used for *Trichoderma* spp. isolation from soil proved to be relatively ineffective for their isolation from *A. bisporus* compost due to the different content of microorganisms that require a different range of inhibitors to exclude them [45]. In order to suppress bacterial growth, different antimicrobials are added into the basic medium; however, attention should be paid to the right choice of antimicrobials as some of them may also suppress hyphal growth or cause the inhibition of conidial germination.

Williams et al. [45] developed a selective medium for the quantitative isolation of *T. harzianum* from champignon compost, which even enables comparisons between aggressive and non-aggressive *T. harzianum* groups. The addition of antimicrobial chloramphenicol, streptomycin, quintozene and propamocarb supported viable hyphae and conidia while suppressing microbial contaminants. In addition, some *Trichoderma* biotypes whose sporulation is triggered or stimulated by the host mycelium, such as *T. aggressivum* f. *europaeum*, might be difficult to reproduce under artificial conditions and without *A. bisporus* [12]. To make the situation even more complicated, there are many *Trichoderma* species populating compost and substrate, and not all of them are aggressive [9]. New species are continuously identified in other cultivated mushrooms, such as *T. hengshanicum* on *G. lingzhi* reported by Cai et al. [32]. It is often impossible to differentiate them from existing species using the culture-based method since they share the same cultivation conditions and/or have very similar macro- and microscopic characteristics. This makes the identification of green mold causative agents by traditional methods very challenging.

## 3. Secondary Metabolite-Based Methods

In the early stage of infection with parasitic fungi *Trichoderma* spp., the compost for *A. bisporus* production shows no signs of disease for up to two weeks after inoculation [11]. During this time, the material is routinely checked for *Trichoderma* presence only if it is a part of the farm’s quality management or if its presence is suspected. Still, it can be a hunt in the dark since there are no standardized procedures for sampling. Some *Trichoderma* spp. appear in raw materials, while others develop only in compost [13], meaning that both raw materials and compost must be checked. Moreover, culture-based methods are meticulous, long and often uncertain, as explained in the previous section.

*Trichoderma* spp., similar to other fungi, produce secondary metabolites, including volatile organic compounds (VOCs), which could be exploited for early *Trichoderma* detection, and some of them have an antibiotic activity [11]. Lee et al. [46] screened 20 *Trichoderma* species (two originated from mushroom compost) and their volatile compounds and identified 141 specific VOCs of different types, including carbohydrates, alcohols, ketones, aldehydes, alkanes, alkenes, esters, aromatic and heterocyclic compounds and terpenes. Different authors reported that *T. aggressivum* strains (DAOM 222156, IMI 393970, IMI 284726) synthesize volatile metabolites such as 6-pentyl-pyrone that stimulate plant growth and reduce disease symptoms [46,47,48]. Krupke et al. [11] discovered that an aggressive North American *Trichoderma* biotype known as Th4 (*T. aggressivum* f. *aggressivum*) produces volatile metabolites that inhibit *A. bisporus* growth. These compounds were different from those produced by the less aggressive biotype Th1. Employing thin layer chromatography, two compounds were identified for the Th4 biotype, one appearing after five days of incubation and the other after 15 days. However, both mentioned compounds were discovered in liquid cultures, while *Trichoderma*-infected compost contained only the second compound. The first compound was identified as 3,4-dihydro-8-hydroxy-3-methylisocoumarin (mellein), while the second has not been characterized. The toxicity of the second compound is such that if it is produced and released too early, it would kill the host (*A. bisporus*) before it degrades compost materials to the level that can be exploited by *Trichoderma*. Thus, the production of toxic metabolites comes in a time-dependent manner when the compost has been degraded to an optimal level and parasitic mycelium is already well spread through the compost. However, Baars et al. [49] showed that the volatile blend of compost infected with *T. aggressivum* can be distinguished from uninfected compost after seven to ten days of spawn run. This finding seems promising for the indirect detection of *T. aggressivum* in compost.

## 4. Overview of Available Molecular Detection Methods

Available molecular methods potentially applicable for detection and monitoring of *Trichoderma* species/strains in edible mushroom production and their main features are summarized in Table 1.

The growth dynamic of *Trichoderma* species in soil and similar complex environments can be successfully tracked using immunological assays. Immunoassays have long been considered fast and sensitive detection methods based on the application of specific monoclonal antibodies (MAbs) to quantify *Trichoderma* biomass in soil [50]. Hybridoma technology allows the production of MAbs that are specific to individual genera, species or even isolates of fungi [51]. Coupled with enzyme-linked immunosorbent assays (ELISA) or immunofluorescence, MAbs can be employed to detect fungi in complex environments [50]. In previous studies, MAbs have been used to detect, quantify and visualize the saprotrophic growth of pathogens in artificially and naturally infested soils [52,53,54,55], to quantify the effects of *T. harzianum* on the saprotrophic growth dynamics of *Rhizoctonia solani* in compost-based systems [56] and for the detection and visualization of *Trichoderma* species in soil or other complex matrices containing mixed fungal populations [57]. In all these studies, an extracellular antigen (enzyme) that is constitutively expressed or could be induced is recognized as an ideal candidate for the MAb-based detection of fungi in soils and composts [57].

Another approach to monitoring the growth dynamics of *Trichoderma* strains is based on genetic modification of the target strains using exogenous markers such as the green fluorescent protein (GFP) gene, hygromycin B phosphotransferase gene, and/or β-glucuronidase encoding gene [50,58,59,60]. Although useful for monitoring targeted strains, this approach is not applicable to the detection of unknown *Trichoderma* strains in a complex environment such as soil or compost.

Diverse fingerprinting molecular techniques are often used for strain- and species-specific detection of *Trichoderma*. The term “DNA fingerprinting” refers to methods that apply DNA digestion with restriction enzymes, PCR amplification, and/or Southern hybridization to generate DNA fragments different in size that can be visualized as specific banding patterns. By comparing banding patterns, it is possible to identify characteristic, strain-specific DNA fragments [50]. The most often used fingerprinting technique for the detection of *Trichoderma* species and strains includes restriction fragment length polymorphism (RFLP), random amplified DNA (RAPD), arbitrary primed PCR (AP-PCR) and universally primed PCR (UP-PCR).

RFLP is a simple molecular technique that uses restriction enzymes to fragment the genomic DNA (gDNA) of an organism. Fragments are electrophoretically separated based on size differences and transferred to the membrane by Southern blotting to create an RFLP pattern. Any two organisms have different RFLP patterns due to differences in the length of fragments produced when the DNA is digested with a restriction enzyme [27].

### 4.1. RAPD-Based Methods

RAPD uses short, usually 10 bp arbitrary primer sequences that can amplify a set of DNA fragments across a targeted genome. This procedure detects nucleotide sequence polymorphisms in a PCR assay without the need for previously determined nucleic acid sequence information. Amplification primers are non-specific, and there is no need for previous knowledge of the target genome sequence. The annealing temperature in PCR amplification is set up to be low, allowing primers to hybridize on multiple mismatched positions across the genome. RAPD amplicons can be analyzed by agarose gel electrophoresis or DNA sequencing if primers are labeled with fluorescent dyes [61,62]. The major limitation of the RAPD method is reproducibility. Low intra-laboratory reproducibility is a consequence of very low annealing temperatures causing a non-specific reaction, leading to the amplification of any contaminating DNA. The method is sensitive to reaction conditions (subtle differences in reagents, protocols and machines), which are the main reasons for low inter-laboratory reproducibility [61,62]. RAPD is widely used in *Trichoderma* spp. studies [16,27,28,30,31,63,64] because it can provide information on genetic variability between different isolates that can be applied to identify diagnostic DNA fragments used for the design of sequence-based, strain-specific markers.

The AP-PCR method is very similar to the RAPD method. RAPD and AP-PCR were independently developed by Williams et al. [65] and Welsh and McClelland [66] in 1990. Several technical details are different in the AP-PCR and RAPD protocols. In AP-PCR, (a) the amplification is conducted in three parts, each with its own stringency and concentration of components, (b) high primer concentrations are used in the first PCR cycles, and (c) primers of variable length and often designed for other purposes are used. Consequently, the advantages and limitations of AP-PCR are identical to those of RAPD [61].

The UP-PCR method is another DNA fingerprinting method similar to the RAPD technique. As for RAPD, with UP-PCR, it is possible to amplify DNA from any organism without previous knowledge of DNA sequences and to generate multi-banding profiles following gel electrophoresis. UP-PCR has the advantage of higher reproducibility when compared to RAPD as it relies on relatively high annealing temperatures, fast ramping, and longer primers. Additionally, the UP-PCR banding profiles consist of higher numbers of bands than most RAPDs, facilitating the identification of species- and strain-specific markers [67].

DNA fingerprinting methods have limited applications due to their problems with reproducibility; however, they can be used to generate strain-specific fragments that can be sequenced and used for the design of species- and/or strain-specific primers for conventional and real-time PCR (RT-PCR)-based strategies. Thus, these fragments, called sequence-characterized amplified regions (SCARs), can be used as monitoring markers [50].

Fingerprinting methods were used to detect pathogenic *Trichoderma* fungi that cause green mold disease in edible mushrooms and to help their taxonomic identification and subsequent description. Using RFLP analysis of ribosomal DNA (rDNA) and mitochondrial DNA (mtDNA), RAPD analysis, as well as internal transcribed spacer 1 (ITS1) sequences, Muthumeenakshi et al. [27] analyzed *T. harzianum* isolates from mushroom compost in the British Isles. Both RFLP and RAPD analyses separated *T. harzianum* into three distinct groups, Th1, Th2 and Th3 (Table 2), with Th2 being the most aggressive. The grouping was further confirmed based on nucleotide sequences of rDNA-ITS1 that generated three ITS sequence types corresponding to RAPD and RFLP groups. This data suggested that *T. harzianum* is a species aggregate that should be separated into three different, molecular-based species. The Th4 group of isolates was detected a few years later in North America and was similar to the Th2 group from the British Isles based on molecular data. The two groups shared the same RAPD profile, while ITS-based RFLP analysis (ITS-ribotyping) and variation in known plasmid types were inconsistent and did not show a correlation with geographic location [28]. The same study recognized the Th3 group as a strain of *T. atroviride,* and this was confirmed by Ospina-Giraldo et al. [15], who used the term biotype to designate the groups. The authors showed that the Th3 biotype clustered together with strains of *T. atroviride* on a phylogenetic tree using ITS1, ITS2 and *5.8S* rRNA gene sequences. Biotypes Th2 and Th4 showed the highest sequence similarity among the green mold-associated biotypes, although they could be readily distinguished from each other, while Th1 was resolved as their closest relative [15]. Using the same approach (ITS1*-5.8S-*ITS2 sequences) on 81 *Trichoderma* spp., Ospina-Giraldo et al. [16] also indicated that green mold-causing biotypes and biocontrol isolates are very closely related and share the most recent common ancestor, although they constitute different phylogenetic groups.

While all previously mentioned studies deal with green mold disease in *A. bisporus*, the disease is not limited to this host only. *Trichoderma* taxa that cause green mold in edible mushrooms were found in fruiting bodies and growing substrates of *A. bisporus* (button mushroom), *A. bitorquis* (pavement mushroom), *Calocybe indica* (milky white mushroom), *Ganoderma lucidum* (lingzhi mushroom), *Lentinula edodes* (shiitake mushroom), *Pleurotus sajor-caju* (oyster mushroom), and *Volvariella volvacea* (paddy straw mushroom). Based on a comparison of ITS1*-5.8S-*ITS2 sequences with NCBI using the BLAST search tool, these taxa were identified as *T. harzianum* and *T. virens* with 98–100% identity, while RAPD analysis exhibited inter- and intra-species variability [64]. However, it is not clear from the article if the authors considered *T. harzianum* as biotype Th1 or as a species aggregate containing biotypes Th1–Th4 (Table 2).

### 4.2. Species-and Strain-Specific PCR

For the screening of pathogenic European biotype 2 (Th2) and North American biotype 4 (Th4), Chen et al. [30] developed a specific PCR-based test using the RAPD-PCR approach. Using primer 232 for RAPD-PCR, an 1150-bp long DNA product that was unique to isolates of Th4 was detected. This product was absent in isolates belonging to the Th1, Th2 and Th3 (*T. atroviride*) biotypes. The 1150-bp DNA product was cloned using *Escherichia coli,* and the cloned DNA was sequenced to obtain sequence data for the design of Th-F and Th-R primers. Primer set Th-F/Th-R amplified an approximately 450-bp DNA product from isolates of Th4 and Th2, while no products were generated with gDNA from isolates of Th1 and Th3 or from several biocontrol *Trichoderma* species. The fact that the PCR-amplified products for Th2 and Th4 were the same size and had a high sequence homology would suggest that at least one of the RAPD-PCR priming sites flanking the 1150-bp DNA product was absent from Th2, although the sequence defined by the primer set Th-F/Th-R resided in both genomes. This test has been especially important because it has the potential to detect Th2 and Th4 specifically, even in complex mixtures of DNA, without the need for pure culture isolation.

For the specific detection of *T. harzianum* strains, Miyazaki et al. [69] developed a molecular assay based on nested PCR. The authors developed primers (THITS-F1 and LR1-1 for the first step and G-THITSF2 and CAA-THITS-R3 for the second step) based on ITS sequences of *T. harzianum* specific strains. By adjusting the annealing temperature and using nested PCR, they developed a specific and highly sensitive assay with a detection limit of *T. harzianum* DNA of 50 fg.

As green mold disease in oyster mushrooms can be caused by diverse *Trichoderma* species leading to significant yield loss, it was important to develop an efficient protocol for *Trichoderma* spp. detection both in mycelial cultures and environments. The multiplex PCR assay has been developed for the identification of *T. pleuroti* and *T. pleuroticola*, the two *Trichoderma* spp. pathogenic to *P. ostreatus* (oyster mushroom). Three oligonucleotide primers (FPforw1, FPrew1 and Psrev1) were designed using sequences within the fourth and fifth introns in the translation elongation factor 1α gene. The PCR assay results in two major bands (447 bp and 218 bp products) for *T. pleuroti* and only the larger fragment for *T. pleuroticola* strains. The assay enables pathogen detection from the substrate samples without the need for isolation and culturing, rendering it useful for the detection of pathogenic fungi during the early phases of infection [70].

*Trichoderma* spp.-specific primers TDP-F/TDP-R were designed using ITS1*-5.8S-*ITS2 sequences of eleven *Trichoderma* strains (two strains of *T. harzianum*, three strains of *T. pleuroticola*, and one strain of *T. longibrachiatum*, *T. atroviride*, *T. koningii*, *T.* cf. *virens*, *T. pleuroti*, and *T. citrinoviride*). A single amplicon was produced for each of the analyzed *Trichoderma* species, and there was no cross-reactivity with the edible mushrooms, nor with *Aspergillus* and *Penicillium*, which are also green molds, but with weak pathogenicity [71]. A similar approach was used a few years earlier to develop a species-specific primer set for four strains of pathogenic fungi (*T. koningiopsis*, *Phomopsis* sp., *Mucor circinelloides* and *Cladosporium bruhnei*) isolated from liquid culture (cultivated in polypropylene bottles) of diseased *P. eryngii* (king oyster mushroom). A species-specific primer set was designed for each fungus from the ITS1*-5.8S-*ITS2 sequences [72].

*Trichoderma* species- and strain-specific primers are also suitable for use in the RT-PCR approach. RT-PCR, also known as quantitative PCR (qPCR), is a technologically advanced PCR-based method that allows the accurate quantification of a target DNA. RT-PCR uses fluorescent probes (TaqMan) or DNA dyes (SYBR Green I dye) to measure the intensity of a fluorescent signal proportional to the quantity of DNA generated during the PCR amplification. This method can be used for direct pathogen detection in an environmental sample [73].

O’Brien et al. [74] applied RT-PCR to specifically detect *T. aggressivum* in bulk phase III substrate for *A. bisporus* cultivation by targeting the translation elongation factor 1α (*TEF1*) gene sequence.

Rubio et al. [73] developed strain-specific primers for *T. harzianum* strain 2413. SCAR A1 and SCAR A1c primers, designed from a previous study involving RAPD analysis of *T. atroviride* 11 [75], were used to test the amplification of DNA from 27 strains of *Trichoderma* spp. The primer set amplified a 1.5 kb fragment in *T. harzianum* 2413. The sequence of the obtained fragment was used for the design of a new primer pair, BR1 and BR2, that amplifies the 837-bp fragment unique to *T. harzianum* 2413. The 837-bp fragment was further used for the design of RT-PCR primers, Q2413f and Q2413r, for specific real-time detection of *T. harzianum* 2413 in sterile and nonsterile soil samples.

### 4.3. Single Locus Sequence Typing and DNA Barcoding

Most fungal species are characterized by relative morphological simplicity, and it is often difficult to identify species based only on morphological features. Thus, DNA barcoding has special importance for fungi. The internally transcribed spacer rRNA region (ITS), including ITS1 and ITS2 spacers separated by the *5.8S* rRNA gene, has been accepted by the International Fungal Barcoding Consortium as the main marker for fungal DNA barcoding. However, some other molecular markers are also in use, especially in cases where the ITS region does not provide sufficient resolution and when there is heterogeneity between ITS copies in the genome [76,77]. Some of the additional or secondary barcoding markers are the intergenic spacer (*IGS*), β-tubulin II (*TUB2*), DNA-directed RNA polymerase II largest (*RPB1*) and second largest (RPB2) subunits, *TEF1*, DNA topoisomerase I (*TOP1*), phosphoglycerate kinase (*PGK*), and cytochrome c oxidase subunit I (*COX1*) and subunit II (*COX2*) [78,79,80,81,82,83,84,85,86,87,88,89,90,91,92,93,94].

Since 2005, several identification tools based on DNA barcoding have been developed for *Trichoderma* spp. TrichOKey was based on full ITS (ITS1-*5.8S*-ITS2) sequences applicable for the quick identification of 75 single species, 5 species pairs, and 1 species triplet of *Trichoderma* described until 2005 [95]. The authors of TrichOKey also developed TrichoBLAST, the toolbox which is a combination of multilocus databases of phylogenetic markers, a diagnostic program for phylogenetic markers (TrichoMARK), and a local BLAST server [96]. These tools were *Trichoderma*-specific, but they were exploited in a moment when the known diversity of *Trichoderma* was around 100 species. Nowadays, a public database with a broad scope of organisms, including fungi, that is mostly used in DNA barcoding studies is the NCBI GenBank (https://www.ncbi.nlm.nih.gov/genbank/, accessed on 15 September 2022), while among the numerous curated databases involving fungal DNA barcoding sequences, the User-friendly Nordic ITS Ectomycorrhiza (UNITE) database is the most popular [97]. In addition, a multilocus identification system for *Trichoderma* (MIST) based on combined information from three DNA barcodes (ITS, *TEF1*, *RPB2*) covering 349 currently known *Trichoderma* species has been developed in 2020 [98].

Although ITS is the official DNA barcode for fungi, *Trichoderma* species identification is mostly based on a combination of different molecular markers. This applies to the *Trichoderma* species causing green mold disease in cultivated mushrooms as well.

Using sequences of the ITS1 region and *TEF1*, the aggressive biotypes Th2 and Th4 are taxonomically separable from *T. harzianum* and described as two forms of the new species, *T. aggressivum* f. *europaeum* and *T. aggressivum* f. *aggressivum* (Table 2). They are morphologically distinguished from *T. harzianum* and *T. atroviride* most readily by their rate of growth [29]. *Trichoderma pleuroti* and *T. pleuroticola*, fungal species associated with green mold disease in oyster mushrooms, were finally described by Park et al. [35]. Their descriptions were supported based on previous phylogenetic analysis of the ITS, the *TEF1* and *RPB2* sequences [99,100].

## 5. Methods Based on High-Throughput Sequencing

After the substantial use of classical Sanger methodology, the next-generation sequencing methodologies started to develop in the second half of the 2000s, enabling high-throughput sequencing (HTS) analyses of fungal communities. In the last several years, even third-generation sequencing methodologies have been developed and applied [101]. HTS marked the beginning of the genomic era and resulted in an ever-rising number of full genome sequences of diverse organisms, including *Trichoderma*. The rapidly increasing number of whole-genome sequences enabled comparative genomic studies to identify exogenous markers for *Trichoderma* species and strain detection [50]. Once identified, trait-specific genetic markers can be developed for the rapid and early screening of strains. Therefore, comparative genomics enables the analysis of the genetic basis that differentiates fungal species/strains [102].

HTS also enables the in-depth study of the environmental sample composition and community composition of microorganisms using metagenomics and metabarcoding approaches. Metagenomics is based on the sequencing of the entire genomes of all microorganisms present in a complex sample. The DNA is extracted from a bulk sample and used for sequencing the collective genomes. The method is suitable for the identification of pathogens and therefore is a precise diagnostic tool that can be used for *Trichoderma* detection [103]. However, it requires considerable resources, as well as relevant knowledge in bioinformatic analysis, that can often be very time-consuming.

**Figure 1:**
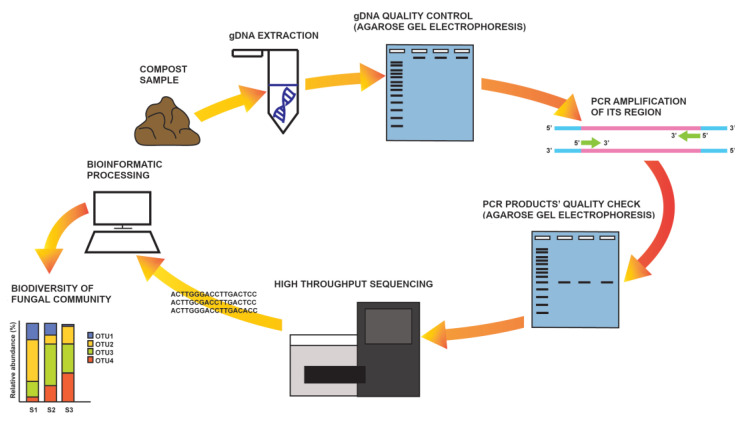
DNA metabarcoding—schematic representation of a workflow.

A somewhat less complicated approach compared to metagenomics is DNA metabarcoding (Figure 1), where only a DNA barcode, i.e., the ITS region in fungi (although some others are also in use), is targeted for HTS in an environmental sample [50]. The procedure includes the PCR amplification of the desired molecular marker and the sequencing of all the amplicons that are representative of all organisms present in the sample. Raw sequence data are first processed bioinformatically, and then sequences are clustered based on their homology and used for taxonomic or functional assignment by comparison to databases [103]. As HTS technologies are highly sensitive, one of the critical steps in the analysis is an experimental design based on multiple replicates. Additionally, special care should be taken to avoid sample contamination and/or sample overgrowing during storage (it is recommended to freeze or dry samples for storage). The DNA extraction procedure should be optimized depending on the sample type (e.g., soil, water, plant tissue). The selection of markers and primers is also critical, as most of them are not suitable for all fungal groups [101].

The second-generation platforms (SOLiD, Roche 454, Illumina, and Ion Torrent) are limited to shorter fragments. They are suitable for metabarcoding based on either the ITS1 or ITS2 region, as the whole ITS (ITS1-*5.8S*-ITS2) is too long to be fully sequenced [101]. Conversely, third-generation sequencing platforms of Pacific Biosciences (PacBio; RSII, Sequel and Revio instruments) and Oxford Nanopore Technologies (ONT; MinION, GridION, and PromethION instruments) are suitable for longer 30- to 50-kb reads on average. However, third-generation methodologies suffer from a high error rate that can be 5 to 20% [104,105,106,107,108].

During the last couple of years, new, improved protocols based on third-generation sequencing that reduce error rates have been developed. The quality of the ONT- and PacBio-generated sequences comparable with Sanger sequencing have been demonstrated in a study based on the amplification of a complete ribosomal operon of authentic fungal herbarium specimens (*Basidiomycota*), aquatic chytrids (*Chytridiomycota*) and one lineage of early diverging fungi (*Nephridiophagidae*) [109]. In 2018, Heeger et al. [110] developed an analysis pipeline based on PacBio circular consensus sequencing that reduced error rates to levels comparable to short-read second-generation platforms. They used a 4500 bp marker containing most of the eukaryote SSU and LSU rRNA genes and the complete ITS region for the DNA metabarcoding of fungi from aquatic environmental samples. Tedersoo et al. [106] evaluated PacBio’s RSII and Sequel instruments for metabarcoding fungi, oomycetes and other eukaryotes in soil samples using full-length ITS barcodes and longer rRNA gene amplicons up to 3000 bp. They concluded that PacBio sequencing is the best method for the metabarcoding of organisms that are of relatively low diversity, require a long barcode sequence for identification, or if phylogenetic analysis should be performed. Loit et al. [111] compared the performance of MinION (ONT) and Sequel (PacBio) instruments for the identification and diagnostics of fungal and oomycetes pathogens from conifer *(Pinaceae*) needles and potato (*Solanum tuberosum*) leaves and tubers. They showed the high performance of the Sequel instruments for the metabarcoding of complex samples to resolve community diversity. On the other hand, MinION can be utilized for the rapid and accurate identification of dominant pathogenic organisms and other associated organisms from plant tissues following both metabarcoding and metagenomic approaches. The authors even developed a rapid diagnostic protocol for the metagenomic approach using MinION that takes only 2.5 h (from sample preparation to bioinformatics result interpretation). However, they conclude that MinION is not suitable for diversity analyses of the whole fungal and oomycetes communities due to its high error rate and multiple biases. A year later, Latorre-Pérez et al. [112] demonstrated that the high error rate of ONT can be overcome depending on the performance of chosen software for sequence assembly. This work demonstrates that despite the high intrinsic error rate of third-generation sequencing platforms, nanopore data alone can overcome these limitations and retrieve extremely contiguous genomes directly from simple microbial communities if appropriate assembly software is used (~99.5 to 99.8% of consensus accuracy).

## 6. Isothermal Nucleic Acid Amplification Methods

### 6.1. Loop-Mediated Isothermal Amplification (LAMP)

**Figure 2:**
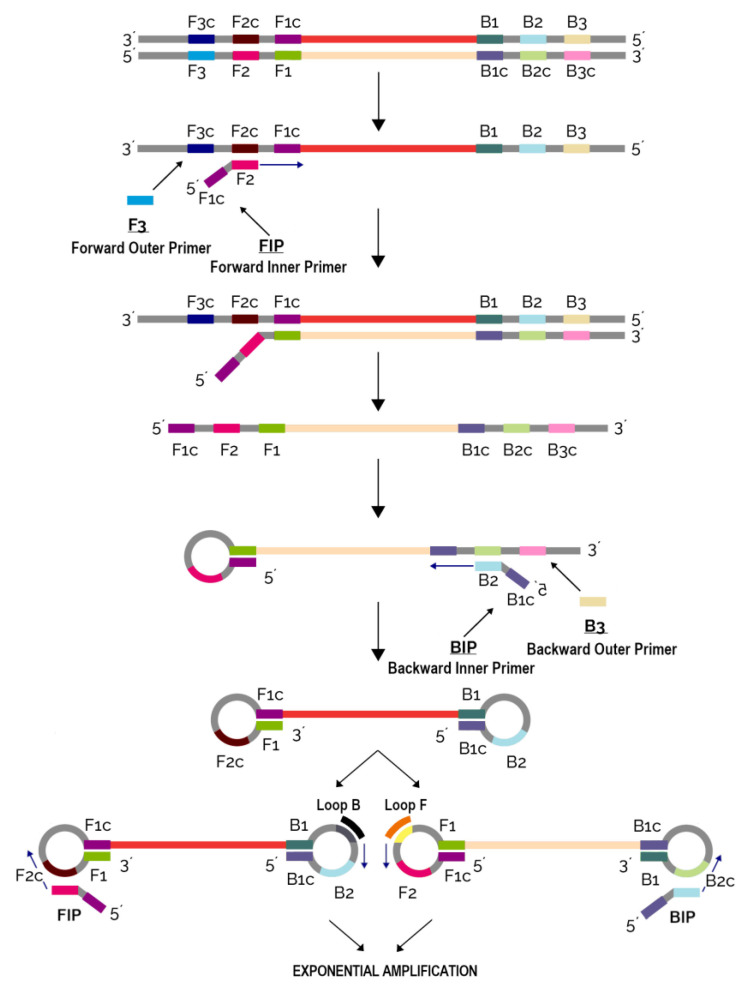
Nucleic acid amplification using loop-mediated isothermal amplification method (LAMP).

The LAMP method developed by the Japanese company Eiken has gained much popularity in the past years in the field of pathogen detection, especially in PON diagnostics [113]. Contrary to conventional PCR techniques that utilize different working temperatures enabled by a thermal cycler, LAMP does not require changing temperatures and hence can be performed by relatively simple, portable and handheld devices. An additional difference to PCR is that LAMP requires a set of four (to six) primers binding to six (to eight) different regions of the target gene. The use of a high number of primers increases reaction specificity (Figure 2). The ease of use, high sensitivity and specificity render LAMP a method of choice for PON diagnostics that can yield fast and precise results from even a small sample, e.g., from only six DNA copies in the reaction mixture [114].

LAMP is an extremely adaptable method that enables the monitoring of multiple reactions in real-time directly in the reaction tube without the need for follow-up analyses, thereby reducing the risk of contamination. Importantly, RT-LAMP, i.e., LAMP containing reverse transcriptase (RT) enzyme in the mastermix, can be used for the direct detection of RNA samples without the need for a separate machine for reverse transcription, i.e., the synthesis of cDNA that is needed for RT-PCR. Another advantage of LAMP compared to some other molecular techniques is the fact that this method is quite resistant to reaction inhibitors that are often found in environmental samples [113,114].

To date, there are not many publications focused on the use of LAMP for fungi. Vaagt et al. published in 2013 one of the very first articles on LAMP used for the identification of mushroom species [115]. In the following years, more publications on LAMP as a tool for the molecular identification of fungi appeared, such as that reported by He et al. [116] on the use of LAMP to distinguish different lethal *Amanita* species. They proved that LAMP can be a time-efficient, cost-effective, specific, and sensitive detection and identification tool. In line with these applications, LAMP has been tested for its potential as a molecular diagnostic tool for the detection of mold infestation in edible mushroom production. For instance, Hu et al. showed that LAMP technology can be used for the in-field monitoring and evaluation of the risk of development of *Botrytis cinerea* resistance to QoI fungicides [117].

Niessen et al. [118] developed a *GAOA* gene (galactose oxidase)-based LAMP assay for the detection of *Fusarium graminearum*, a species that produces different mycotoxins and causes *Fusarium* head blight of small grain cereals. This specific and time-saving LAMP assay was shown to detect less than 2 pg of purified DNA per reaction within 30 min [118]. Similarly, Li et al. [119] developed an easy, rapid (DNA amplified in 60 min), sensitive and highly specific LAMP assay for the detection of *F. oxysporum* f. sp. *cubense* race 4 (Foc race 4), a fungus that causes a banana disease known as *Fusarium* wilt (Panama disease). The assay is based on a SCAR marker sequence, can be used in the field, and has a detection limit of 10 fg per 25 μL reaction in pure culture [119]. Another example of a successful LAMP application was reported by Luo et al. [120] for species of the genus *Aspergillus*, which are known to produce carcinogenic aflatoxins and pose a risk to consumer safety. These authors designed three sets of LAMP primers to identify *A. nomius*, *A. flavus* and *A. parasiticus* and could specifically detect these pathogenic species in coffee beans, peanuts and Brazil nuts. Furthermore, Niessen et al. [121] provided one of the first reviews of the LAMP method used for the detection of mycotoxigenic fungi and spoilage yeasts in different food samples.

### 6.2. Recombinase Polymerase Amplification (RPA)

RPA is another isothermal nucleic acid amplification method that has become widespread in a short period due to its simplicity, speed, and sensitivity [122]. An RPA reaction is performed by using 32–36 nucleotide length primers at 37–42 °C for 5–20 min (depending on the initial number of copies and the size of the amplicon). The three main components of an RPA reaction are: (1) enzyme recombinase (usually *E. coli* RecA), (2) single-strand binding (SSB) proteins and (3) DNA polymerase (most commonly Sau DNA polymerase from *Staphylococcus aureus*) [123]. The RPA reaction is very fast; the reaction starts as soon as the sample is in contact with the reagents, and it is not necessary to use high temperatures to denature the DNA molecules. Specifically, the first enzyme recombinase examines the dsDNA target to find the homologous primer-binding site and open up the double-helix structure, which is then stabilized by the SSB proteins. It is important to note that this all occurs in the presence of ATP molecules. The recombinase can be further decomposed by ATP hydrolysis, after which polymerase replaces the strand to connect complementary nucleotides to the primer sequence to form a new DNA strand. RPA allows the amplification of very short fragments of nucleic acids, as well as 1–2 kb long fragments, which cannot be performed with any other isothermal technique [124,125,126]. However, the application of RPA is somewhat limited by the fact that there is only one provider of the RPA reagents—the UK company TwistDxTM.

Karakkat et al. [127] designed LAMP and RPA assays for root-infecting fungal pathogens *Gaeumannomyces avenae*, *Magnaporthiopsis poae* and *Ophiosphaerella korrae*. Both methods proved to be fast (results obtained in 30 min) and efficient (they involved minimal sample preparation). However, the authors’ opinion is that the RPA assay was more effective because it had less false-positive contamination compared to the LAMP assay [127]. Sakai et al. established a visible microarray system and conducted an RPA for the labeling and amplification of pathogenic DNA products in order to identify pathogenic fungal DNA at the species level [128]. In the study conducted by Roumani et al. [129], RPA coupled with a lateral flow (RPA-LF) assay was developed for the detection of spoilage fungi in fruit-based products. The method has been shown to be extremely sensitive (can detect down to 1.2 pg/µL of pure fungal DNA) and has a low limit of detection (LOD) (45.7 spores/50 g and 1.0 CFU/50 g for molds and yeasts, respectively). This fast and efficient assay for fungi detection can be adapted for PON applications [129].

An efficient RPA-based method has been recently developed for rapid on-spot detection of *Aspergillus flavus* in grains. This assay showed great rapidity, efficiency, and potential for application in many other fields [130].

### 6.3. Nucleic Acid Sequence Based Amplification (NASBA)

NASBA is an isothermal transcription-based amplification system specially designed for RNA detection. It was first described by Compton in 1991 [131]. NASBA is used for the continuous amplification of nucleic acids under isothermal conditions, usually at 41 °C. Traditional methods of detecting RNA molecules involve the use of the RT-PCR technique, a long-term process that often results in false-positive results due to cross-contamination [132].

Additionally, none of the known isothermal amplification methods can amplify RNA molecules directly with such a rate of sensitivity as NASBA. It allows the amplification of more than 109 copies of target RNA within only 30 min by the functioning of three enzymes: Avian Myeloblastosis Reverse Transcriptase (AMV-RT), RNase H, and RNA polymerase [132].

It is also important to highlight that NASBA results in single-stranded RNA (ssRNA) amplicons. Such products provide an opportunity for another round of amplification without the need for previous strand separation [133].

However, the greatest benefit is that NASBA can selectively amplify RNA even in the presence of genomic DNA, making this method extremely useful for analyzing complex samples such as compost and casing soil previously used in the diagnostics of parasitic fungi in mushroom production [132,134].

## 7. Clustered Regularly Interspaced Short Palindromic Repeats (CRISPR)

The CRISPR/Associated Protein (Cas) system was discovered in bacteria during the 1980s [135]. The system uses Cas enzymes and a guide RNA (gRNA) to cleave target single-strand (ss) DNA, double-strand (ds) DNA or ssRNA. There are two main classes of CRISPR/Cas systems depending on the number of effector subunits that makes Cas activity. Class I systems use a multimer Cas effector complex, while class II uses single, RNA-guided, multidomain Cas proteins. Each of the classes contains multiple types and subtypes of CRISPR/Cas systems. The most used are (1) type II systems such as Cas9, (2) type V with subtypes Cas12 and Cas14 (designated now as Cas12f), and (3) type VI with subtype Cas13 [136]. Cas enzymes differ in their activities and use the gRNA of different structures. CRISPR/Cas9 recognizes dsDNA, CRISPR/Cas12a recognizes dsDNA and/or ssDNA, CRISPR/Cas13 targets ssRNA and CRISPR/Cas14 targets ssDNA. Cas12a, Cas13, and Cas14 also exhibit collateral non-specific activities against ssDNA or ssRNA (Cas13) and, in an activated state, cleave not only the target DNA or RNA but also any DNA or RNA in the environment [136,137]. Additionally, CRISPR/Cas can also be used for the detection of non-nucleic acid targets, including proteins, analytes, and hormones (for details, see Bhardwaj et al. [138]).

The biosensing application of CRISPR/Cas systems is most often coupled with nucleic acid amplification methods and especially isothermal methods such as RPA and LAMP that additionally increase their sensitivity [138]. *Fusarium* head blight (FHB), a disease of wheat caused by the pathogenic fungus *F. graminearum*, can be rapidly detected in an early phase using a CRISPR/Cas12a-based nucleic acid assay. The assay is based on the highly specific recognition of PCR amplicons of the ITS and *EF1α* of *F. graminearum* by the gRNA. This triggers the collateral activity of the Cas12a protein, and it cleaves reporter ssDNA probes with terminal fluorophore and quencher groups, resulting in fluorescence signal detection. The assay can detect 1 fg/μL total DNA from *F. graminearum* that corresponds to fourth-day infection [139]. Additionally, the RPA-CRISPR/Cas12a system was established with the integration of a lateral flow assay (LFA) readout system for the diagnosis of citrus scab (*Elsinoë fawcettii*), a fungal pathogen of citrus. The system is highly sensitive (a minimum amount of 1 fg of *E. fawcettii* gDNA without cross-reactions for non-scab fungal pathogens can be detected), cost-effective and rapid (within one hour). It is also suitable for crude DNA extract analysis and can be conducted at a relatively low temperature (37 °C), which makes it suitable for in-field applications [140].

The CRISPR/Cas system is mostly used in *Trichoderma* for gene editing in order to develop novel engineered strains for desired applications in industry and agriculture, given their lignocellulose degradability and biocontrol activities [141]. To our knowledge, there is no published research where CRISPR/Cas systems were used for pathogen *Trichoderma* species and strain detection, although they possess considerable potential.

## 8. Conclusions

In this review, we described molecular methods for the monitoring and detection of pathogenic fungi in environmental samples. Some of them have been previously applied in *Trichoderma* spp., and others were reported for other fungi but may have great potential for *Trichoderma* green mold diagnosis. We can distinguish between these methods by considering those that are culture-dependent and require previous isolation of *Trichoderma* species/strains present in the sample (DNA fingerprinting methods and DNA barcoding) and those that are suitable for direct *Trichoderma* screening (RT-PCR, HTS methods, CRISPR/Cas and nucleic acid isothermal amplification methods). Culture-dependent methods are time-consuming and suitable for laboratory applications. The direct methods are highly specific and sensitive but not necessarily rapid. HTS methods are often time-consuming, laboratory intensive, and demand advanced data processing skills. However, due to the wide range of applications, there are efforts to define standardized and simplified protocols for sample analysis and data processing. The equipment used for HTS methods is usually highly sophisticated and linked to the laboratory. The only exception is MinION, the third-generation Oxford Nanopore Technology portable device that can be used for metagenomics and metabarcoding analyses in-field. For real-time detection of *Trichoderma* spp., CRISPR/Cas and nucleic acid isothermal amplification methods seem the most promising since they are fast, accurate, highly specific, highly sensitive, and unlike RT-PCR, can be adapted for in-field applications, i.e., they have the potential to be implemented in the PON devices.

The real-time detection of *Trichoderma* during early infection is essential for the prevention and control of the spread of green mold disease in edible mushroom production. Therefore, in addition to strict hygiene and treatments with disinfectants as a first step in green mold disease prevention, the early screening of mushroom-growing substrate should be adopted as a part of a regular control procedure. The screening should be applied repeatedly during the mushroom cultivation process since the absence of infection in the first period does not imply its absence during the later stages of mushroom growth. Regular screening will enable the timely application of treatments and decrease the application of aggressive chemicals as the most efficient against green mold. This practice will give space to alternative, environmentally friendly treatments, such as the microbial biocontrol of green mold disease.

## Figures and Tables

**Table 1:** Evaluation of available molecular methods potentially applicable for detection and monitoring of *Trichoderma* species/strains in edible mushroom production.

Method			T	S	SE	RE	EQ	PON
	Immunoassays		2	b	+	+	+	−
	Exogenous markers		2	a	+	+	+	−
	DNA fingerprinting	RFLP	3	a, b	−	−	+	−
DNA-based		RAPD	2	a, b	−	−	+	−
		AP-PCR	2	a, b	−	−	+	−
		UP-PCR	2	a, b	−	−	+	−
	Species- and strain-specific PCR		2	a, b	+	+	+	−
	Single locus sequence typing and DNA barcoding		3	b	+	+	+	−
	HTS	DNA metabarcoding	4	b, c	+/−	+/−	+	+/−
		Metagenomics	5	b	+/−	+/−	+	+/−
	CRISPR/Cas		1	a, b	+	+	+/−	+
	Isothermal nucleic acid amplification	LAMP	1	a, b	+	+	−	+
		RPA	1	a, b	+	+	−	+
		NASBA	1	a, b	+	+	−	+

T, time consumption (1 = up to one hour, 2 = one to six hours, 3 = six to twelve hours, 4 = 12 h to 72 h, 5 = more than 72 h); S, specificity (a = strain-specific, b = species-specific, c = genus-specific); SE, highly sensitive; RE, highly reproducible; EQ, needs sophisticated equipment; PON, suitable for point-of-need application; (+) applicable; (−) not applicable; (+/−) depends on the specific method/equipment used.

**Table 2:** Main *Trichoderma harzianum* green mold-associated biotypes found in *Agaricus bisporus* cultivation.

Biotype	Species	Reference
Th1	*Trichoderma harzianum*	Rifai [68]
Th2	*Trichoderma aggressivum* f. *europaeum*	Samuels et al. [29]
Th3	*Trichoderma atroviride*	Castle et al. [28]; Ospina-Giraldo et al. [15]
Th4	*Trichoderma aggressivum* f. *aggressivum*	Samuels et al. [29]

## Data Availability

No new data were created or analyzed in this study. Data sharing is not applicable to this article.
